# Association between social capital and depression among critically ill patients: evidence from a cross-sectional study in rural Shandong, China

**DOI:** 10.1186/s12888-021-03476-9

**Published:** 2021-09-27

**Authors:** Yaru Zhang, Jiajia Li, Feng Fang, Wenhao Fu

**Affiliations:** 1 Centre for Health Management and Policy Research, School of Public Health, Cheeloo College of Medicine, Shandong University, Jinan, 250012 China; 2 NHC Key Lab of Health Economics and Policy Research (Shandong University), Jinan, 250012 China

**Keywords:** Social capital, Critically ill patients, Depression, Rural China

## Abstract

**Background:**

With an increasing number of critically ill patients, attention should be paid to both their physical health and mental health. The objective of this study is to examine the links between depression and social capital among critically ill patients.

**Methods:**

Data for 1043 patients with critical illnesses was collected with a stratified cluster random sampling method in rural Shandong, China. Depression symptoms were measured using a short form version of the Center for Epidemiologic Studies Depression Scale (CESD-10) and the total scores of them were dichotomized. We associated structural social capital with social networks, social participation, and social support. Cognitive social capital includes the degree of availability of social trust and reciprocity. Binary logistic regression was used to explore whether social capital was significantly associated with depression among patients with critical illnesses.

**Results:**

We found that 68.5% of the critically ill patients in our sample population had depression. CESD-10 scores were negatively correlated with social capital, including occupations of their frequent contacts, social trust in relatives and friends, distance to the nearest medical institution and medical assistance convenience from non-spouse. In addition, low economic status, and low self-rated health were more significantly correlated with depression in critically ill patients.

**Conclusions:**

Our findings suggest that more attention should be paid to the mental health of critically ill patients and more formal society, community and government support form given, particularly in rural China.

## Background

Critical illnesses, such as cancer, refractory diseases, etc., usually refers to diseases that are typically costly, long-lasting, difficult to cure, and significantly affect the productivity and quality of life of patients and their families [1]. To prevent families of critically ill patients from falling into catastrophic health expenditure (CHE) [2], the Chinese government introduced the Critical Illness Insurance in 2012 and defined critical illnesses as one in which annual out-of-pocket medical expense exceeds a certain threshold, which is usually set with reference to local disposable income [3]. Critical illnesses are growing increasingly common globally, for example, cancer rates have increased, with new cases of cancer ranged from 14.9 million to 18.1 million, and their associated death toll ranging from 8.2 to 9.6 million between 2013 and 2018 [4, 5].

There has been increasing recognition that social capital has a positive effect on health outcomes, not only physical health, but also mental health [6, 7]. For instance, Coll-Planas found that social capital had a protective influence on health, furthermore, interventions based on the social capital had achieved wonderful results on the health of the elderly [8]. Ehsan hold the view that individual cognitive social capital is negatively associated with common mental disorders [9]. In Bassett’s study, neighborhood social capital reduced the likelihood of depressive symptoms among urban-dwelling adults [10]. Moreover, previous researchers concluded that social networks, and social participations promoted health and decreased depression [11, 12].

Social capital’s demonstrable effect on people’s mental health has been well documented in previous studies [9, 13]. However, there is also a group of people whose psychological problems and reduced social capital as a result of critical illness deserve our attention. For one thing, patients with critical illnesses suffered from long-term diminished quality of life [14], daily functioning and long periods of stay at home or in hospital [15], which resulted in having narrow social networks cycle and social participation [16]. For the other thing, depression is usually a co-morbidity for critical illnesses, as the pain, disappointment and helplessness caused by cancer may result in low psychological resilience or psychological impairment [17–19]. Nevertheless, both the government and society typically focus more on the economic burden and physical health of critically ill patients, and less to their declining mental health and social capital.

Previous studies have shown that, cancer patients who have good interpersonal relationships with others, were less depressed than that with poor relationships, which were linked to low-level social support [20, 21]. As an extended concept of social determinants of mental illness [9], social capital measures the quality and quantity of social relationships [22], will provide us with better evidence on how to promote mental health at the societal level. For patients with critical illnesses whose social capital is lower than that of the general population, there are few theoretical and empirical studies on whether social capital is still a protective factor of mental health. Our study attempts to partially fill this gap.

In addition, this study pays special attention to rural patients in China. Compared with urban areas, rural China has scarce medical human and material resources which increased the prevalence of critical illnesses in part to the unavailability of timely treatment, low utilizations of medical services [23], and imperfect community characteristics [24], although it also boasts closer relationships between relatives and neighbors [25]. However, with the process of urbanization, the phenomenon of hollowing out has become increasingly serious in rural regions [26], and the originally close interpersonal relationships has been gradually destroyed. According to the data from National Bureau of Statistics, unbelievably, the urban population of the total population has ranged from 18% in 1978 to 61% in 2019 [27]. Most young people choose to live or work in cities, the remaining older, large “empty-nesters” population, are more vulnerable to both critical illnesses and depression due to age, loneliness and their relatively lower family support [28, 29]. Data from China’s 12th Five-Year Plan for the Development of Aging illustrated that, the empty-nest rate of the elderly in rural China was 38.3%. The proportion of empty-nest elderly households is expected to reach 90% by 2030 [30]. As the population shrinks and ages rapidly, rural interpersonal relationships based on geographical location and blood has been hit hard [31]. Family size shrinking, interpersonal networks decreasing, family pension weakening made the elderly remained in rural areas lonely and depressed [29].

Accordingly, our study investigated the relationship between both structural and cognitive social capital and depression among patients with critical illnesses and explored ways in which they might improve their mental health and quality of life.

## Methods

### Participants and procedures

The participants chosen for this study were limited to critically ill patients in rural Shandong, China between July and August, 2019. Two inclusion criteria for critical illnesses in our research are as follows: 1) With reference to the Critical Illness Insurance Policy of Shandong Province in 2019, critical illnesses were identified as diseases with high out-of-pocket (OOP) expenses which exceeded the local critical illness insurance reimbursements threshold (The threshold is 12,000 to 16,000 RMB in sample areas of Shandong province). 2) Although annual OOP not up to the reimbursement threshold, diseases with long treatment cycle (more than 2 years), low cure rate and high total medical expenses, such as sequela of stroke, hematopoietic stem cell transplantation, and end-stage renal disease, are also defined as critical illness. In order to avoid research bias, 2 samples with mental illness or non-disease caused disabilities have been excluded from the data of this study.

Three representative cities from the eastern, central, and western regions of Shandong Province were selected to provide sampling variety. We used a stratified cluster random sampling method in our surveys, accounting for factors such as socioeconomic development, medical resource availability, demographics, and geography. Interviewers conducted door-to-door visits and face-to-face interviews with participants. After removing invalid or incomplete data, the total sample consisted of 1043 patients with critical illnesses from 77 villages. We ensured that the questionnaires were only used for data analysis and protected patient privacy.

These studies were approved by the Ethics Committee of School of Public Health, Shandong University.

### Measurements

#### Depression

The dependent variable of our study was the depression symptoms of patients with critical illnesses, which we quantified with the 10-item Center for Epidemiologic Studies Depression Scale (CESD-10) from China Health and Retirement Longitudinal Study (CHARLS). This survey, an abbreviated but more experimentally effective variant of the 20-item CESD developed by Radloff [32], offers a numeric value to assess patients’ relative depression. The CESD-10 asks subjects to rate each response in terms of the frequency that each mood or symptom occurred “during the past week” on a Four-Point scale [33], scoring 0 (‘< 1 day’), 1 (‘1–2 days’), 2 (‘3–4 days’) and 3 (‘5–7 days’). Two positive questions on the CESD-10 are ‘I felt hopeful about the future’ and ‘I was happy’, the point options of which are 3, 2, 1, and 0, respectively. According to the recommendations of Andresen [33], a total score ≥ 10 (out of a maximum of 30) indicates that the patient has depressive symptoms, and a total score < 10 indicates that the patient is not depressed. The CESD-10 is reliably internally consistent (Cronbach alpha = 0.872). Additionally, CESD-10 data is spherically distributed (KMO = 0.909, *P* < 0.001) and thus suitable for factor analysis. Confirmatory Factor Analysis (CFA) has successfully tested the construct validity of the CESD-10 scale. In addition, it is generally believed that the Tucker-Lewis Index (TLI) > 0.9 as well as both the Root Mean Square Error of Approximation (RMSEA) and the Standardized Root Mean-square Residua (SRMR) < 0.08 indicate construct validity for the CESD-10 (TLI = 0.964; RMSEA = 0.060; SRMR = 0.0295).

#### Social capital

Social capital theory, derived from the intersection of economics and sociology, has been developing for a long time. A number of sound studies have explored the conceptualization of social capital [34–37]. Social capital has a broad definition and subsequently varies and even conflicted between disciplines [36]. Some of them are theory-near while others are easy to measure and use proxies [36, 37]. To serve the research objectives, the definition of social capital in this paper is mainly referenced from the sixth edition of the Dictionary of Epidemiology, which is operational and easy to measure within health survey [38]. In addition, we draw upon the seminal works of relevant studies on mental health and social capital [9, 39, 40], as well as social capital of Chinese population [39, 41]. We conceptualize social capital as the resources available and chosen by individuals—for example, trust and norms of reciprocity; and the resources that are embedded within an individuals’ social networks—for example, social support, social participation, and community networks.

**Fig. 1:**
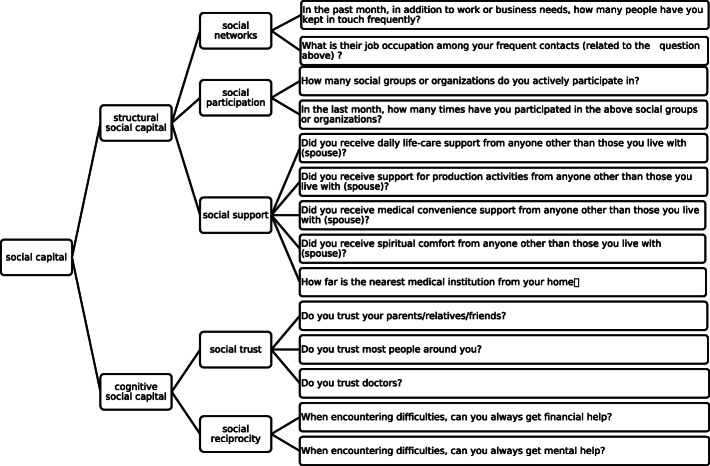
Framework and explanations of social capital variables

To measure social capital, we follow the “structural /cognitive” distinction that is widely recognized and used [8, 9, 40, 41], to reflects two features of social capital: the quantity and quality of social interactions [9, 42]. In our study, cognitive social capital consists of social reciprocity and social trust, while structural social capital includes social network, social participation and social support (see Fig. 1).

What needs illustration is that we place social support within an extended social capital framework, although some of the literatures has treated social capital with social support as two separate concepts [9]. The reasons are as follows. First, according to the definition of social capital in the sixth edition of the Dictionary of Epidemiology [38], “The resources—for example, social support, information channels, social credentials—that are embedded within an individuals’ social networks” are a component of social capital. Of the studies included in Rodgers’s updated review from 2007 to 2018, 34% studies have considered social support as a part of social capital [7]. Especially when we look at the relationship between social capital and mental health, social capital is strongly correlated with access to social support, both of which can act as a buffer against mental disorders [9, 43, 44]. Second, for patients with critical illnesses, whether they can obtain social support is an important indicator reflecting the quality of social network, because they are in real need of social support [45].

Social reciprocity and social trust were assessed via ‘totally agree’ and ‘other’ responses. Occupations of frequent contacts were divided into ‘none’, ‘farmer’ and ‘other’ categories. ‘None’ indicates that patients do not have frequent contacts. Social support to patients with critical illnesses, which consists of daily life-care support, producing activities assistance, medical convenience and spiritual comfort, was categorized as ‘spouse’ and ‘other’. ‘Other’ indicates that anyone other than those you live with (spouse), including neighbors or friends, relatives, and children or parents.

#### Social demographic characteristics

Demographic factors included in this study were: gender (Male, Female), age (in years), education (in years), marital status (Married, Other), living status (Live alone, Empty nest, Other), economic status (Economic surplus, Sufficient means, Having difficulties), number of diseases (Number of critical illnesses), and self-rated health status (on a scale of 1 to 5, with lower ratings indicating greater health). As shown in the Table 1, age, education, number of disease and patients’ self-rated status were continuous variables while gender, marital status, living status, economic status were categorical variables.

**Table 1:** T-test and chi-square test of depression

Variable	Not Depressed n (%)	Depressed n (%)	***t/χ***^***2***^
**Gender**			6.19*
Male	177 (35.26)	325 (64.74)	
Female	152 (28.10)	389 (71.90)	
**Age [Mean (SD)]**	61.37 (13.77)	63.43 (11.76)	−2.48*
**Education [Mean (SD)]**	5.73 (3.54)	5.12 (3.47)	2.62**
**Marital status**			2.57
Married	291 (32.48)	605 (67.52)	
Other	38 (25.85)	109 (74.15)	
**Living status**			3.25
Live alone	19 (24.05)	60 (75.95)	
Empty Nest	168 (30.83)	377 (69.17)	
Other	142 (33.89)	277 (66.11)	
**Economic status**			56.64***
Economic surplus	73 (48.99)	76 (51.01)	
Sufficient means	177 (36.80)	304 (63.20)	
Having difficulties	79 (19.13)	334 (80.87)	
**Number of diseases [Mean (SD)]**	1.13 (0.40)	1.14 (0.39)	−0.36
**Self-rated health status [Mean (SD)]**	2.88 (0.81)	3.63 (0.77)	−14.39***
**Social networks**			
**Number of frequent contacts [Mean (SD)]**	6.74 (6.20)	4.86 (5.05)	5.21***
** Occupations of frequent contacts**			21.93***
None	5 (7.94)	58 (92.06)	
Farmer	195 (30.71)	440 (69.29)	
Other	129 (37.39)	216 (62.61)	
**Social participation**			
**Number of group involvement [Mean (SD)]**	0.26 (0.45)	0.12 (0.35)	5.34***
**Number of group activities participated in the last month [Mean (SD)]**	0.59 (1.14)	0.23 (0.75)	6.02***
**Social reciprocity**			
**Economic help in need**			63.00***
Agree totally	181 (46.29)	210 (53.71)	
Other	148 (22.70)	504 (77.30)	
**Mental help in need**			59.80***
Agree totally	206 (43.83)	264 (56.17)	
Other	123 (21.47)	450 (78.53)	
**Social trust**			
**Trust in relatives and friends**			39.26***
Agree totally	263 (37.95)	430 (62.05)	
Other	66 (18.86)	284 (81.14)	
**Trust in most people around**			26.16***
Agree totally	209 (38.63)	332 (61.37)	
Other	120 (23.90)	382 (76.10)	
**Trust in doctors**			8.59**
Agree totally	259 (34.12)	500 (65.88)	
Other	70 (24.65)	214 (75.35)	
**Social support**			
**Daily life-care support**			0.44
Spouse	125 (32.81)	256 (67.19)	
Other	204 (30.82)	458 (69.18)	
**Producing activities assistance**			1.34
Spouse	117 (33.91)	228 (66.09)	
Other	212 (30.37)	486 (69.63)	
**Medical convenience**			0.56
Spouse	75 (29.64)	178 (70.36)	
Other	254 (32.15)	536 (67.85)	
**Spiritual comfort**			0.05
Spouse	104 (32.00)	221 (68.00)	
Other	225 (31.34)	493 (68.66)	
** Distance to the nearest medical institution [Mean (SD)]**	0.54 (0.45)	0.61 (0.53)	−1.94

*Note*: Statistical description of variables. Continuous variables use mean and standard deviation (SD), categorical variables use frequency (n) and percentage. Standard deviation (SD) or percentage (%) is in parentheses

Sample size:1043

*P < 0.05 **P < 0.01 *** *P* < 0.001

### Data analysis

All statistical analysis was performed by using Stata version 14.0 and R software. We conducted univariate analysis to test the effect of independent variables and control variables on depression as defined by CESD-10 scores. CESD-10 values were assessed as dependent variables and dichotomized. Continuous variables and categorical variables were analyzed by using t-test and chi-square tests respectively. Significant factors identified from univariate analysis were analyzed with a logistic regression model to investigate their odds ratios (ORs) and 95% confidence intervals (Cis) [46]. Some control variables and independent variables served as dummy variables to make the regression result indicate a positive explanation. In order to achieve the optimal regression result, we adjusted and screened variables continuously. In statistical inference, *P* < 0.05 indicates that the variation is statistically significant (two-sided).

## Results

### Sample description

Frequency and percentage were used to describe categorical variables, and the mean and standard deviation (SD) of variables were provided to summarize continuous variables (see Table 1). Natural logarithmic transformation was performed on continuous variables that have a maximum influence, such as number of group activities participated in the last month and distance to the nearest medical institution. For patients who were depressed, the mean age (SD) was 63.43 (11.76) years, the average education duration (SD) was 5.12 (3.47) years, 54.48% were females, 52.80% were empty-nesters and 53.50% expressed social distrust.

### Univariate analysis results

As shown in Table 1, demographic characteristics analyzed with respect to depressed critically ill patients included gender, age, education, economic status, self-rated health status. Structural and cognitive social capital had varying relationships to depression.

### Binary logistic regression results

Table 2 summarizes the binary logistic regression results that show the relationships between depression and structural and cognitive social capital.

**Table 2:** Two-level logistic regression results of depression

Variable	Depressive symptom
	***OR*** [95%***CI***]
**Control variables**	
**Gender**	
Female (ref: Male)	1.33 [0.96, 1.83]
**Age**	1.00 [0.99, 1.02]
**Education**	1.02 [0.97, 1.07]
**Marital status**	
Married (ref: Other)	0.94 [0.49, 1.79]
**Living status**	
Live alone (ref: Other)	1.36 [0.58, 3.19]
Empty Nest (ref: Other)	1.07 [0.74, 1.54]
**Economic status**	
Economic surplus (ref: Having difficulties)	0.58 [0.36, 0.95] *
Sufficient means (ref: Having difficulties)	0.54 [0.38, 0.78] **
**Number of diseases**	0.91 [0.60, 1.38]
**Self-rated health status**	2.69 [2.17, 3.35] ***
**Independent variables**	
**Social networks**	
**Number of frequent contacts**	0.98 [0.95, 1.01]
**Occupations of frequent contacts**	
Farmer (ref: None)	0.31 [0.11, 0.88] *
Other (ref: None)	0.30 [0.10, 0.88] *
**Social participation**	
**Number of group involvement**	1.10 [0.57, 2.10]
**Number of group activities participated in the last month**	0.83 [0.62, 1.10]
**Social reciprocity**	
**Economic help in need**	
Agree totally (ref: Other)	0.64 [0.41, 1.00]
**Mental help in need**	
Agree totally (ref: Other)	0.89 [0.55, 1.44]
**Social trust**	
**Trust in relatives and friends**	
Agree totally (ref: Other)	0.61 [0.39, 0.94] *
**Trust in most people around**	
Agree totally (ref: Other)	1.23 [0.82, 1.85]
**Trust in doctors**	
Agree totally (ref: Other)	0.75 [0.51, 1.10]
**Social support**	
**Daily life-care support**	
Other (ref: Spouse)	0.85 [0.51, 1.40]
**Producing activities assistance**	
Other (ref: Spouse)	1.25 [0.72, 2.17]
**Medical convenience**	
Other (ref: Spouse)	0.55 [0.31, 0.98] *
**Spiritual comfort**	
Other (ref: Spouse)	1.46 [0.89, 2.40]
** Distance to the nearest medical institution**	1.45 [1.04, 2.01] *

*Note*: Odds ratios (*OR*) reported and 95% credible interval (*CI*) in parentheses

Sample: 1043

*P < 0.05 **P < 0.01 ***P < 0.001

Most aspects of social capital were negatively associated with depression, although some were not statically significant. With respect to social networks, critically ill patients who had frequent contacts with farmers or others had a significant negative correlation with depression (farmer *OR* = 0.31, 95%*CI*: 0.11 to 0.88, *P* = 0.028; other occupations *OR* = 0.30, 95%*CI*: 0.10 to 0.88, *P* = 0.028). Moreover, a negative correlation was found between patients with low trust in relatives and friends and depressive symptoms (*OR* = 0.61, 95%*CI*: 0.39 to 0.94, *P* = 0.026). When it comes to social support, distance to the nearest medical institution, had a positive relationship with depressive moods among patients (*OR* = 1.45, 95%*CI*: 1.04 to 2.01, *P* = 0.027). We also found that if spouses provided medical convenience for patients had a negative effect on depression (*OR* = 0.55, 95%*CI*: 0.31 to 0.98, *P* = 0.041). However, the impact of other variables of social capital on depression proved to be statistically non-significant.

With respect to control variables, patients with critical illnesses who had economic difficulties had significantly higher odds ratios of depression than patients who did not have economic burdens (Economic surplus *OR* = 0.58, 95%*CI*: 0.36 to 0.95, *P* = 0.031; Sufficient means *OR* = 0.54, 95%*CI*: 0.38 to 0.78, *P* = 0.001). Age, gender and education, however, did not play any significant role in levels of depression. In addition, self-rated health status had a significant association with depressive moods (*OR* = 2.69, 95%*CI*: 2.17 to 3.35, *P* < 0.001). Remarkably, however, marital status, living status as well as the number of diseases a patient suffered from were likewise not significantly associated with depression.

## Discussion

Many studies have explored the mental health of the elderly or mental illness unilaterally, but few concentrated on the impact of social capital on mental health among critically ill patients. Our study found that 68.5% of patients with critical illnesses were depressed, a population higher than that of the general population [47, 48]. Our sample was somewhat unusual (patients with critical illnesses) and depression understandably has greater incidence in patients who experience long-term physical impairments and a reduced quality of life [12, 33, 49]. Along similar lines, one earlier study suggested that patients with critical illnesses are prone to heart failure due to decreased body function and greater vulnerability to underlying diseases [50], a finding that may be extrapolated to patients’ mental health.

Here, we consider the relationship between social capital and depression for patients who suffering from critical illnesses. For one thing, about structural social capital, frequent contacts within social networks were negatively correlated with depression. This implied that frequent contacts interacted with patients and provided them with both material and emotional support. In our study, 76.0% patients who lived alone and 92.1% patients who had no frequent contacts were depressed, confirming previous findings that loneliness, social isolation and living alone are all risk factors for depression and a leading cause of mortality [51]. For another, the reasons why social participation was not significantly associated with depression may be there are few opportunities for critically ill patients to participate in formal or informal social activities (such as mahjong, chess, cards, or community sports participation) due to physical impairments [52]. In our sample study, the social participation rate was only 16.0%. Previous findings demonstrated that 14 countries have carried out social participation intervention activities to improve the mental health of critically ill patients, such as social skills training and supported community engagement, even offering patients economic incentives for participation [53].

From the perspective of social support, patients whose homes were far from the nearest medical institutions were more vulnerable to depression because they could not have timely treatment for their illnesses, lowering their recovery rate. We found that by reducing depression among critically ill patients, social capital also lowered medical support requirements. Although great progress has been made in access to health services in rural areas since China’s new medical reforms and its promotion of the ‘15 minutes medical circle’ construction initiative, a gap remained between the capacities of medical services in urban and rural areas [54]. Additionally, psychological interventions are difficult to implement, because there were rarely skilled psychological and guidance counselors in township health centers and village clinics with high medical demands [55], or more generally in communities with high patient density [56]. Besides, China clinical psychology has yet to catch on within primary medical institutions or indeed the rural residents themselves [55]. As things stand, insufficient attention is paid to patients’ mental health. But for many patients, there are more practical issues with care availability. Indeed, our research suggests that only 24.9% patients enjoyed high levels of medical convenience and that 35.9% received daily life-care support from spouses. One reason why spouses supplied medical convenience to critically ill patients was a risk factor is that spouses not only must take care of patients and their parents, but also assume the financial burdens and reduce social activities, which lead to depression and anxiety that can negatively influence critically ill patients [57]. Paradoxically, the support provided by the spouse is the most effective among close relationships at lowering stress in Kang and Han’s study [58]. Medical support, critical illness insurance and measures such as China’s New Cooperative Medical System (NCMS) can relieve the anxiety and depression among patients. Patients with critical illness insurance and NCMS attach more importance to health risk factors and choose healthy lifestyles [59].

When it comes to cognitive social capital, the effect of mental help, as well as financial aid, proved to be statistically non-significant, which was unexpected. With respect to our findings, 80.9% of patients with economic difficulties were depressed. Except for individual cognitive social capital, neighborhood social capital may influence collective mental health, and a deficit of such social capital may have an impact on self-rated health and psychological distress [60]. In contrast to the above, spiritual comfort enhances individual well-being, which, in turn, may reduce the risk of unhealthy behaviors [61]. Finally, interpersonal trust, or individual trust in relatives and friends, reduces social complexity and helps to build strong and sincere social networks [62]. 76.1% of our study’s critically ill patients who had low levels of trust in others generally, and 75.4% of patients who had low trust in doctors were depressed that was inconsistent with other researchers’ findings [16], which may link to the poor capacities of village doctors. Though Chinese medical competences have improved nationally, rural China lags behind. Indeed, only 15% of doctors in township health centers and 2.3% of doctors in village clinics had a bachelor degree or above [63].

Differentiated with previous findings in the general population [64], gender was not a risk factor for depression in our findings. Moreover, 65.6% of patients were aged 60 and over in our study, and the prevalence of critical illnesses among the elderly is continuously increasing owing to population aging which calls for more insight into the factors that contribute to their mental health [65]. China will have a significant elderly population by 2022, and this population will grow at rates exceeding those of Sweden or France [66]. Accordingly, attention must be paid to the mental health of the elderly, especially for those suffering from critical illnesses. Our research also showed that, contrary to traditional thinking [67, 68], education, living status, number of diseases have nothing to do with the patients’ depression. Self-rated health status is a subjective indicator, and often used to describe patients’ health status, which is similar to the CESD scale.

Our research has some limitations. First, our survey is cross-sectional, which precludes us from identifying causal relationships between depression and social capital. Second, there is no specific scale or theoretical framework for social capital, in which biases measurement. Third, our research objects were critically ill patients, so we cannot know whether the social capital of patients with critical illnesses is different from that of the general population.

## Conclusions

Encouragingly, social capital including occupations of frequent contacts in social networks, social trust in relatives and friends, distance to the nearest medical institution, and spouse-provided medical conveniences in social support play a positive role in depression. Due to functional disabilities and relatively low social capital, patients with critical illnesses are more likely to have depression than others.

Looking to the future, effective measures should be taken to improve the quality of life and mental health among critically ill patients. Patients can be encouraged to be optimistic about all aspects of their lives via frequent communication. Family members should call more, go home more often, and supply both necessary financial support and spiritual comfort for patients. Alternatively, it is essential to strengthen mental health services and set up specific personnel such as rural doctors or psychologists to provide health education and psychological counseling to patients. Social participation interventions, like social skills training and supported community engagement [53] should be implemented. Additionally, we propose that governments increase funding for rural medical resources to reduce urban-rural disparities and health inequity, strengthen training of grassroots medical staffs, and make primary care medical insurance more accessible. Otherwise, group-based education and social support programs aiming to prevent social isolation by improving community knowledge and networks are meaningful, such as focus-group discussions or city tours [69]. In summary, our communities, families, and individuals each need to provide a greater degree of both medical support and spiritual comfort to critically ill patients.

## Data Availability

The datasets used and analysed during the current study are available from the corresponding author on reasonable request.

## Abbreviations

CESD: Center for Epidemiologic Studies Depression Scale
CESD-10: 10-item Center for Epidemiologic Studies Depression Scale
CHARLS: China Health and Retirement Longitudinal Study
NCMS: China’s New Cooperative Medical System
CI: Confidence Interval
OR: Odds ratio
CHE: Catastrophic Health Expenditure
